# ELDER ABUSE AND THE OPIOID CRISIS: PERPETRATORS WHO ARE SUBSTANCE USERS

**DOI:** 10.1093/geroni/igz038.2804

**Published:** 2019-11-08

**Authors:** Karen A Roberto, Pamela B Teaster

**Affiliations:** 1 Virginia Tech, Blacksburg, Virginia, United States; 2 Center for Gerontology, Virginia Tech, BLACKSBURG, Virginia, United States

## Abstract

Substance abuse, particularly the diversion/abuse of prescription drugs along with illicit opioid deviates by alleged perpetrators has been identified as is a risk factor for elder abuse. The purpose of this study was to characterize cases of elder abuse substantiated by APS in which the perpetrator used opioids and related substances. Guided by the Contextual Theory of Elder Abuse, we conducted a within-case/across-cases thematic analysis of Kentucky APS caseworkers’ notes on 40 substantiated cases of elder abuse. Financial exploitation was the most commonly identified type of abused associated with perpetrators who abuse opioids. Findings revealed that most cases of elder abuse occurred when the perpetrators’ substance abuse intersected with employment status, complex family relationships, and a history of altercations with the law. Findings provide new insights into a more elaborate conception of the ways in which the opioid epidemic is contributing to the perpetration of elder abuse today.

